# Supplementary material for: A Cryptochrome 2 mutation yields advanced sleep phase in humans
Source: eLife. 2016 Aug 16;5:e16695. doi: 10.7554/eLife.16695 (PMC5398888; doi:10.7554/eLife.16695)
Supplement: Figure 1—source data 1. — Subject IDs correspondence to numbers in Figure 1A. DOI: http://dx.doi.org/10.7554/eLife.16695.004 [file elife-16695-fig1-data1.docx]

**Figure1- source data 1. Summary of sleep phenotype of human subjects**

| **Subject ID** | **Age**^a^ | **sDLMO**^b^ | **Sleep log**  **MST** | **Zeo log**  **MST** | **Strctrd Intrvw**  **MST** | **H-O MEQustnr**  **Score** |
| --- | --- | --- | --- | --- | --- | --- |
| 100336 | 49 | 16:41 | 0:59 | 1:00 | 1:32 | 84^c^ |
| 101271s | 49 | N/A | N/A | N/A | 1:11 | >/= 72 |
| 101375 | 79 | N/A | N/A | N/A | 3:15 | 60 |
| 101374 | 21 | N/A | N/A | N/A | 6:52 | 34 |

^a^ age when they were phenotyped

^b^ home saliva-DLMO. Normal human sDLMO: Mean (SD) = 20:50(1:12), Range 18:13 to 00:26, n=163.

^c^ among the top 0.3 % of middle age adults
